# Preparation, Physicochemical and Antioxidant Properties of Acid- and Pepsin-Soluble Collagens from the Swim Bladders of Miiuy Croaker (*Miichthys miiuy*)

**DOI:** 10.3390/md16050161

**Published:** 2018-05-12

**Authors:** Wen-Hao Zhao, Chang-Feng Chi, Yu-Qin Zhao, Bin Wang

**Affiliations:** 1 Zhejiang Provincial Engineering Technology Research Center of Marine Biomedical Products, School of Food and Pharmacy, Zhejiang Ocean University, Zhoushan 316022, China; zhaowenhao166@sina.com (W.-H.Z.); zhaoy@hotmail.com (Y.-Q.Z.); 2 National and Provincial Joint Laboratory of Exploration and Utilization of Marine Aquatic Genetic Resources, National Engineering Research Center of Marine Facilities Aquaculture, School of Marine Science and Technology, Zhejiang Ocean University, Zhoushan 316022, China

**Keywords:** miiuy croaker (*Miichthys miiuy*), swim bladders, acid-soluble collagen (ASC), pepsin-soluble collagen (PSC), physicochemical property, antioxidant activity

## Abstract

Collagen is one of the most useful biomaterials and widely applied in functional food and cosmetics. However, some consumers have paid close attention to the safety of mammalian collagens because of the outbreaks of bovine spongiform encephalopathy (BSE), foot-and-mouth disease (FMD), and other prion diseases. Therefore, there is a strong demand for developing alternative sources of collagen, with one promising source being from the process by-products of commercial fisheries. In this report, acid-soluble collagen (ASC-SB) and pepsin-soluble collagen (PSC-SB) from swim bladders of miiuy croaker (*Miichthys miiuy*) were isolated with yields of 1.33 ± 0.11% and 8.37 ± 0.24% of dry swim bladder weight. Glycine was the major amino acid present, with a content of 320.5 (ASC-SB) and 333.6 residues/1000 residues (PSC-SB). ASC-SB and PSC-SB had much lower denaturation temperatures compared to mammalian collagen, a consequence of low imino acid contents (196.7 and 199.5 residues/1000 residues for ASC-SB and PSC-SB, respectively). The data of amino acid composition, SDS-PAGE pattern, UV and FTIR spectra confirmed that ASC-SB and PSC-SB were mainly composed of type I collagen. FTIR spectra data indicated there were more hydrogen bonding and intermolecular crosslinks in ASC-SB. These collagens showed high solubility in the acidic pH ranges and low NaCl concentrations (less than 2%). The Zeta potential values of ASC-SB and PSC-SB were 6.74 and 6.85, respectively. ASC-SB and PSC-SB presented irregular, dense, sheet-like films linked by random-coiled filaments under scanning electron microscopy. In addition, ASC-SB and PSC-SB could scavenge DPPH radical, hydroxyl radical, superoxide anion radical, and ABTS radical in a dose-dependent manner. Overall, the results indicate that collagens from the swim bladders of miiuy croaker are a viable substitute for mammalian collagen, with potential functional food and cosmeceutical applications.

## 1. Introduction

Collagen is the major structural protein in the connective tissue of vertebrates and constitutes about 30% of the total animal protein [1]. It has a unique triple-helical structure formed by three polypeptide α-chains to form connective tissues and maintain the structural integrity of cells [1,2]. Currently, at least 29 types of collagen (type I-XXIX) have been isolated and identified from a variety of animal tissues, with each type of collagen having its own unique amino acid sequence, molecular structure, and biophysical properties [3]. Collagen possesses many useful features, such as high tensile strength, good biocompatibility, low antigenicity and cytotoxicity, and has found broad applications in food, pharmaceutical, and cosmetic industries [4,5,6]. In the food industry, collagen is widely used as an ingredient to enhance the elasticity, consistency, and stability of food products. Collagen can also be hydrolyzed by proteases to prepare peptides with functions including skin-lightening, antioxidant activity, decreasing blood pressure, and increasing bone mineral density [7,8,9]. Therefore, the global demand for collagen has been continuously increasing over the years, with simple, sufficient, and cheaper supplies of collagen crucially needed.

Traditionally, products from land-based mammals—including pig skin and bovine tendon—have been the major source of collagen [1]. However, the outbreak of bovine sponge encephalopathy (BSE), transmissible spongiform encephalopathy (TSE), foot-and-mouth disease (FMD), and avian influenza have resulted in anxiety among users of collagen and collagen-derived products from land-based animals in recent years [10,11]. In addition, porcine collagen is prohibited in some regions by various religious groups, including Muslims and Jews, due to religious and consumer related issues [5,12]. Therefore, alternative sources of collagen have received increasing attention, including the processing by-products of commercial fisheries, which account for approximately 75% of the total catch weight [1,3,5]. Sun et al. extracted acid-soluble collagen (ASC) and pepsin-soluble collagen (PSC) from Pacific cod, and these were triple-helical type I collagens with a uniform, porous structure, and good biocompatibility [1]. Jeevithan et al. isolated type-II collagens from whale shark (WS) cartilage, and these collagens could be a suitable alternative to mammalian collagen as a biomaterial for commercial application [13]. Coelho et al. prepared collagen from Antarctic and Subantarctic squid, and these collagens showed potential application in hybrid scaffolds for tissue engineering [14]. Therefore, these aquatic by-products are suitable substitutes for mammalian collagen that satisfy not only the requirements of Judaism (kosher) and Islamism (halal), but also alleviate consumers’ concerns about FMD and BSE, and increase economic returns for the fishery industry while reducing environmental pollution [15,16].

Miiuy croaker (*Miichthys miiuy*) belongs to family Sciaenidae and is a commercially important marine fish distributed in Northwest Pacific: from western Japan to the East China Sea [17]. In China, the miiuy croaker is an important commercial fish and aquaculture species, which has been widely cultured since late 1990s because of its fast growth, various feeding habit, medicinal value, and high economic value [17]. Lu et al. prepared the hydrolysates of swim bladder collagen and proved the hydrolysates could enhance learning and memory of mice [18]. However, there was little information available about the extraction of collagen from the swim bladders of miiuy croaker. Therefore, the aim of the present study was to extract and characterize acid-soluble collagen (ASC-SB) and pepsin-soluble collagen (PSC-SB) from the swim bladders of miiuy croaker (*M. miiuy*) to a greater degree than previously done.

## 2. Results and Discussion

### 2.1. Proximate Composition Analysis 

Chemical compositions of swim bladders from miiuy croaker, as well as the ASC-SB and PSC-SC derived from them, are shown in Table 1. The major component of swim bladder was moisture, protein, fat, and ash. The moisture content of swim bladders from miiuy croaker were higher than those from big head carp (75.2%) [19], but lower than those of yellowfin tuna (83.3%) [10] and catla (82.8%) [20]. The protein content of the swim bladders is 1.58-fold higher than those of yellow fin tuna [10]. In addition, ASC-SB and PSC-SB had higher protein contents (94.25 ± 2.57 and 95.02 ± 3.04 g/100 g respectively) than that (74.31 ± 2.66 g/100 g) of swim bladders from miiuy croaker. These data indicated that the extraction process had effectively removed the impurities in swim bladders of miiuy croaker.

Table 1 highlights that the yield of ASC-SB was 1.33 ± 0.11%, which was slightly higher than those of ACS from swim bladders of yellowfin tuna (1.07%) [10], but significantly lower than those of ACS from the swim bladder of seabass (85.3%) [21] and catla (22.2%) [20]. This result suggested that the swim bladder of miiuy croaker might have more cross-linked collagen than that of seabass. Therefore, pepsin was used in effort to increase the yield of collagen of swim bladders of miiuy croaker because it can specifically cleave peptides at the telopeptide region, thereby facilitating the extraction of collagen from the fibrils matrix. As shown in Table 1, the yield of PSC-SB was 8.37 ± 0.24% of the beginning dry weight of swim bladder and was 6.29-fold that of ASC-SB. This result is also in agreement with reports that a limited quantity of pepsin (0.1%) could effectively dissolve collagens from skin of Nile tilapia [1], skin and bone of spanish mackerel [11], spine and skull of skipjack tuna [16], and swim-bladders of grass carp [21] and bighead carp [19]. However, the yield of PSC-SB from swim bladders of miiuy croaker is still lower than those of PSC from swim bladders of yellowfin tuna (12.10%) [10], bighead carp (59.0%) [19], and catla (61.3%) [20]. The differences in yields of ASC and PSC might be attributed to differences in the biological conditions of the aquatic species, with cross-linking of collagen fibrils in the raw material governing the collagen extraction ratio. The present result suggested that there were a high proportion of cross-links in the swim bladders of miiuy croaker and a more effective extraction method for breaking the cross-links in swim bladders should be designed to accelerate the collagen yield.

### 2.2. Amino Acid Analysis

The amino acid composition of type I collagen from calf skin (CSC), ASC-SB, and PSC-SB from swim bladders of miiuy croaker were expressed as amino acid residues per 1000 total amino acid residues (residues/1000 residues) and presented in Table 2. The results indicated that ASC-SB and PSC-SB had similar amino acid compositions, with glycine as the most abundant amino acid. In addition, ASC-SB and PSC-SB were also rich in proline, alanine, and glutamic acid/glutamine, in decreasing order, while negligible amounts of cysteine and tryptophan were also found. The unique amino acids of hydroxyproline and hydroxylysine in collagen were also found in ASC-SB and PSC-SB. In general, glycine represents about one-third of the total residues and is normally spaced at the beginning of typical tripeptide repetitions (Gly-X-Y, where X is mostly proline and Y is hydroxyproline) present in areas of collagen that do not include the first 10 or so amino acids at the C-terminus and the last 14 or so amino acids at the N-terminus [11,19]. In addition, glycine, as the smallest amino acid with only a hydrogen atom side chain, allows the three helical chains to form the final superhelix. The amino acid profiles of ASC-SB and PSC-SB were quite similar to those of type I collagen purified from other aquatic by-products, including swim-bladders of seabass, grass carp, and bighead carp [18,21,22], scales of redlip and croceine croakers [23], and skin of tilapia and spanish mackerel [5,11].

The imino acid (proline and hydroxyproline) contents of ASC-SB and PSC-SB were 196.7 and 199.5 residues/1000 residues respectively, which is higher than those of collagen from swim bladders of yellowfin tuna (ASC: 128 residues/1000 residues, PSC: 169 residues/1000 residues) [10], seabass (ASC: 194 residues/1000 residues) [21], bighead carp (175 residues/1000 residues) [19] and grass carp (ASC: 161 residues/1000 residues, PSC: 157 residues/1000 residues). However, the imino acid contents of ASC-SB and PSC-SB were significantly lower than those of CSC (216.6 residues/1000 residues) and pig skin collagen (220 residues/1000 residues) [24]. Imino acids contribute to the stability of the helix structure of collagen and are one of the key properties determining their potential applications. Pyrrolidine rings of imino acids have been confirmed to impose restrictions on changes in the secondary structure of the polypeptide chain, and hence help to strengthen the thermal stability of triple helical structure [25]. Moreover, hydroxyproline has been verified as playing an important role in stabilizing the triple-stranded helix of collagen by forming interchain hydrogen bonds through the hydroxyl group [8,11]. Therefore, the contents of imino acid are very important for the structural integrity of collagen. As such, the helices of ASC-SB and PSC-SB might be more unstable than those of calf and pig skins collagen due to their lower contents of imino acids.

### 2.3. Electrophoretic Pattern

**Figure 1:**
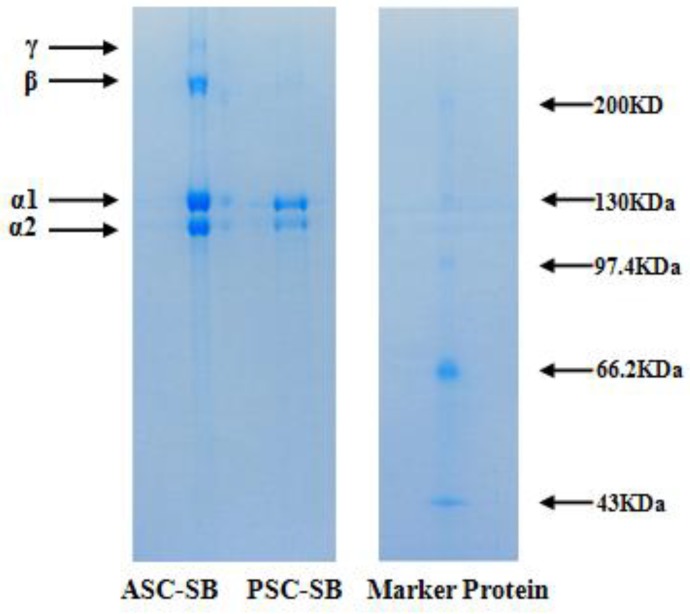
SDS-PAGE patterns of ASC-SB and PSC-SB from swim bladders of miiuy croaker (*M. miiuy*).

Electrophoretic pattern is commonly used to analyze the type and composition of collagen, and is based on the subunit composition, electrophoretic mobility, and intensity of the band. As shown in Figure 1, similar PAGE protein patterns of ASC-SB and PSC-SB were observed. ASC-SB and PSC-SB consisted of two α-chains (α1 and α2) as the major constituents, and the α1 and α2 chains with molecular weight of approximately 115 and 108 kDa, respectively, were found at a ratio of approximately 2:1. The result suggested that ASC-SB and PSC-SB from swim bladders of miiuy croaker were characterized as type I collagen ([α1]_2_α2). The electrophoretic patterns of isolated ASC and PSC were in in agreement with collagens from swim-bladders of yellowfin tuna [10], seabass [21], grass carp [22], and bighead carp [19]. In addition, comparing the SDS-PAGE patterns between ASC-SB and PSC-SB, it could be found that ASC-SB contained the high molecular weight band belonging to the β- and γ-chains, which suggested that the intra- and inter-molecular cross-links of collagens were richer in ASC-SB. For PSC-SB, there were no β- and γ-chains detected, indicating an absence of the cross-linking that is normally contained in the telopeptide region of tropocollagen and that the β- and γ-components were cleaved into α-components by the pepsin [26].

### 2.4. Spectral Properties of Collagen

#### 2.4.1. Ultraviolet (UV) Absorption Spectrum

**Figure 2:**
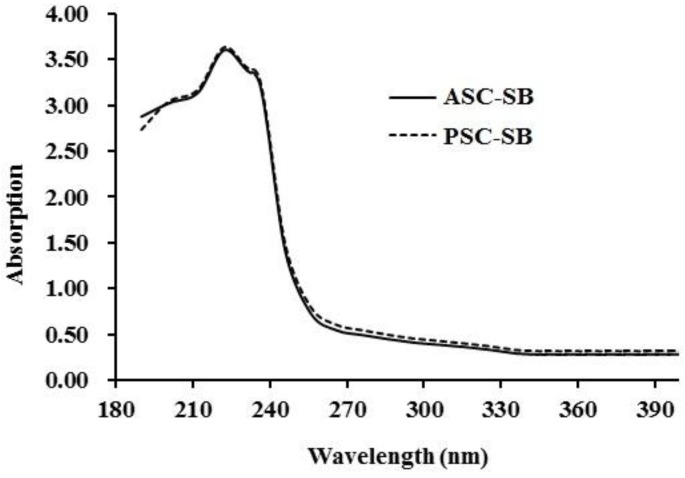
UV spectra of ASC-SB and PSC-SB from swim bladders of miiuy croaker (*M. miiuy*).

Ultraviolet scanning of collagen is one of the simplest methods to evaluate its purity, because the triple helical structure of collagen has a maximum absorption peak in the 210–240 nm range [16,20]. As presented in Figure 2, the UV absorption spectra of ASC-SB and PSC-SB exhibited a maximum absorbance peak at 226 nm, which was similarly reported for collagen isolated from spine and skull of skipjack tuna (*Katsuwonus pelamis*) [16]; swim bladder and scale of carp [20,27]; and skin of catla, barramundi, and tilapia [28,29]. This maximum absorption peak observed between 220 and 240 nm could be related to presence of C=O, −COOH, and CONH_2_ in the polypeptide chains of ASC-SB and PSC-SB. In addition, no distinct absorption peak was recorded at 280 nm, which might be due to the very low amount of aromatic amino acids such as phenylalanine, tryptophan, and tyrosine in ASC-SB and PSC-SB which can absorb UV light at 280 nm [20,27].

#### 2.4.2. Fourier Transform Infrared Spectroscopy (FTIR)

**Figure 3:**
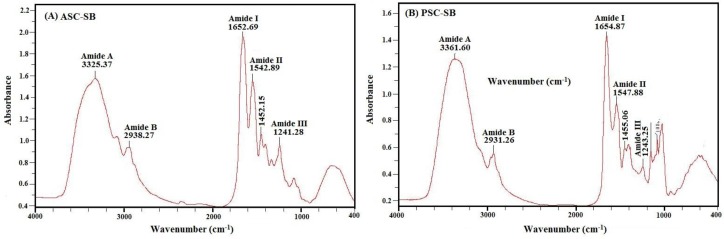
FTIR spectra of ASC-SB and PSC-SB from swim bladders of miiuy croaker (*M. miiuy*).

The representative FTIR absorption spectra (4000–400 cm^−1^) of ASC-SB and PSC-SB from swing bladders of miiuy croakers and the assignment of major peaks (amide A, B, I, II, and II) are shown in Figure 3. The major absorption bands of ASC-SB and PSC-SB were in amide band region, including the peak of amide A (ASC-SB 3325.37 cm^−1^, PSC-SB 3361.60 cm^−1^), amide B (ASC-SB 2938.27 cm^−1^, PSC-SB 2931.26 cm^−1^), amide I (ASC-SB 1652.69 cm^−1^, PSC-SB 1654.87 cm^−1^), amide II (ASC-SB 1542.89 cm^−1^, PSC-SB 1547.88 cm^−1^), and amide III (ASC-SB 1241.28 cm^−1^, PSC-SB 1243.25 cm^−1^). These peaks arise from the vibration of the peptide groups, and provide information about the secondary structure of polypeptides.

The band of amide A is bound up with the N–H stretching frequency. The wavenumber (cm^−1^) of a free N–H stretching vibration is located next to 3400–3440 cm^−1^ range, and the wavenumber would move to lower frequency if the N–H group participated in the formation of a hydrogen bond [30,31]. Figure 3 shows that the amide A wavenumbers of ASC-SB and PSC-SB were 3325.37 cm^−1^ and 3361.60 cm^−1^, respectively. The data illustrated that some N–H groups in ASC-SB and PSC-SB contributed to the formation of hydrogen bonds, and the degree of hydrogen bonding in ASC-SB was more than that of PSC-SB. The amide B band is related to asymmetric stretch vibrations of ${–NH}_{3}^{+}$ and =C–H, and the shift of amide B to higher wavenumber is associated with an increase in free ${NH–NH}_{3}^{+}$ groups from both lysine residues and the N-terminus [11,30]. The wavenumbers of amide B band of ASC-SB and PSC-SB were found at 2938.27 cm^−1^ and 2931.26 cm^−1^ respectively, indicating ASC-SB had fewer free ${–NH}_{3}^{+}$ groups than PSC-SB did.

Amide I and amide II bands are known to be related to the degree of molecular order and to be involved with the triple helical structure of collagen, resulting from C=O stretching, N–H bending, and C–H stretching, respectively [32]. The amide I band is associated with the C=O stretching vibration along the polypeptide backbone or a hydrogen bond coupled with COO– and has strong absorbance in the range of 1600–1700 cm^−1^. The amide I band is a sensitive marker of the peptide secondary structure and a reduction in molecular order will make the peak shift to a lower wavenumber [2]. Figure 3 shows that the amide I band of ASC-SB is 1652.29 cm^−1^, while that of PSC-SB is slightly higher at 1654.87 cm^−1^. This illustrates that the preparation process of PSC-SB using pepsin degrades part of the telopeptides and leads to an absence of the active amino acids (lysine, hydroxylysine, histidine) that requires inter- and intra-molecular cross-linking [33].

The amide II band is primarily responsible for the NH bending with a contribution from CN stretching vibrations, which generally occurs in the range of 1550–1600 cm^−1^. In other words, the amide II band specifies the number of NH groups involved in hydrogen bonding with the adjacent α-chain; therefore, a lower wavenumber of the amide II band indicates both increased hydrogen bonds by NH groups, and a higher structure order [34]. The wavenumbers of ASC-SB and PSC-SB were found to be 1542.89 and 1547.88 cm^−1^, respectively. In addition, Liang et al. reported a shift of amide I and amide II to higher frequencies was associated with an increase in the molecular order [31]. Yousefi et al. suggested that the amide II band of collagen located in low wavenumber had more hydrogen bonds between adjacent α-chains [31]. The present data indicated that there was more hydrogen bonding in ASC-SB.

Amide III absorption, which is associated with the triple helix structure of collagen and involves both C–N stretching and N–H in plane bending from amide linkages, is normally very weak in FTIR. It also arises from wagging vibrations of CH_2_ groups from the glycine backbone and proline side-chains [35]. In this study, the amide III bands of ASC-SB and PSC-SB were located at wavenumbers of 1241.28 cm^−1^ and 1243.25 cm^−1^, respectively. The result indicated that hydrogen bonds were involved in ASC-SB and PSC-SB. In addition, the absorption peaks at 1452.15 cm^−1^ and 1455.06 cm^−1^ were found for ASC-SB and PSC-SB respectively, which corresponded to pyrrolidine ring vibration of proline and hydroxyproline [35]. The intensity ratio between the amide II band and the 1450 cm^−1^ band has been used to elucidate the triple-helical structure of collagen [36], and the absorption ratio between amide III (ASC-SB 1241.28 cm^−1^, PSC-SB 1243.25 cm^−1^) and 1452.15 cm^−1^ (ASC-SB) or 1455.06 cm^−1^ (PSC-SB) bands were approximately equal to 1.0, which confirmed that the triple helices of both ASC-SB and PSC-SB were still intact and a high extent of intermolecular structure was still maintained [36].

### 2.5. Viscosity and the Denaturation Temperature (T_d_)

The stability of the triple helix of collagen depends on interchain hydrogen bonding, side-chain interactions, and the hydration shell. The triple helix structure can be depolymerized and transformed into an unordered coil configuration if the intra-molecular hydrogen bonds are broken by high temperature. This, in turn, results in changes in physical characteristics such as a decrease in solubility, precipitation, and a reduction in viscosity [11,34,37]. Viscometry is a simple and effective method to investigate the conformational change of macromolecules in solution. Therefore, viscosity measurement is often applied to research the thermostability of collagen [10,16].

**Figure 4:**
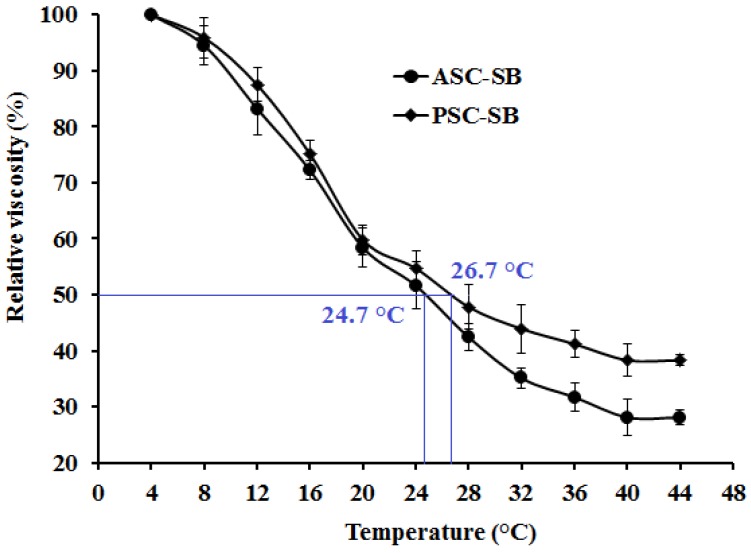
Relative viscosity (%) change of ASC-SB and PSC-SB from swim bladders of miiuy croaker (*M. miiuy*). All data are presented as the mean ± SD of triplicate results.

The relative viscosities of ASC-SB and PSC-SB solutions at the concentration of 0.6% are shown in Figure 4, and a similar rapid decline trend was observed for each when the temperature increased from 4 to 44 °C. The T_d_ values of ASC-SB and PSC-SB were 24.7 and 26.7 °C respectively, which were lower than those of skin collagens from pig and calf (37 °C) [11]. The data further confirmed that the helix structures of ASC-SB and PSC-SB were more unstable than those of collagens from mammals. The T_d_ and viscosity of PSC-SB were slightly higher than that of ASC-SB, which might be in accordance with the higher cross-links of PSC. The presence of imino acids, particularly hydroxyproline in ASC and PSC (196.7 and 199.5/1000 residues respectively), might contribute to the stabilization of the triple helix structure through hydrogen bonding in coiled coil α-chains [10].

### 2.6. Solubility

#### 2.6.1. Effect of NaCl Concentration

**Figure 5:**
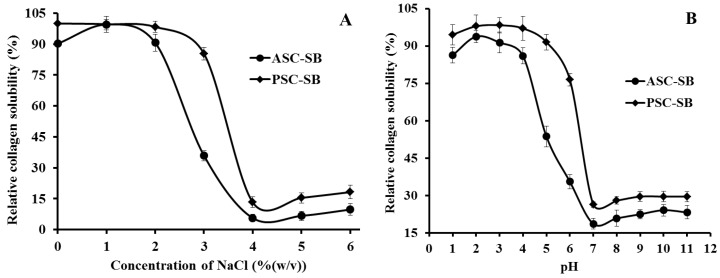
Solubilities of ASC-SB and PSC-SB from swim bladders of miiuy croaker (*M. miiuy*) in 0.5 M acetic acid at different concentrations of NaCl (**A**) and pH (**B**). All data are presented as the mean ± SD of triplicate results.

Figure 5A shows the relationship of solubility and NaCl concentration within a 0–6% range. The results highlight a similar pattern for the solubilites of ASC-SB and PSC-SB, with only slight differences within the tested concentration range. The solubility of ASC-SB and PSC-SB remains high (>90%) when the NaCl concentration was lower than 2%, and rapidly declined as the NaCl concentration increases. The minimum solubility of ASC-SB and PSC-SB is when the NaCl concentration is 4%, after which solubility remains low with further increases in NaCl concentration. The solubility decrease of collagen might be on account of the ‘salting out’ effect resulting from the relatively high NaCl concentrations (>3%). An increasing ionic strength could improve the hydrophobic–hydrophobic interactions of protein chains and increase the competition for water with the ionic salts, which leads to protein precipitation by increasing hydrophobic interactions and aggregation [11,38]. The result was similar to the solubilities of collagens from skin and bone of spanish mackerel [11]; scales and skin of tilapia [5]; fins, scales, skins, bones and swim bladders of bighead carp [19]; skin of channel catfish [38]; and scale of croceine and redlip croakers [23].

#### 2.6.2. Effect of pH

Solubility of ASC-SB and PSC-SB from swim bladders of miiuy croaker in relation to pH is depicted in Figure 5B. The results made clear that ASC-SB and PSC-SB showed the highest solubility at pH 2 (*p* < 0.05), were more easily solubilized at low pH range (1–4), and a sharp decrease in solubility was observed when pH was higher than 4 (*p* < 0.05). In addition, the minimum solubility of ASC-SB and PSC-SB was at about pH 7. After which collagen solubility presented a slight upward trend when the pH was more than 7. Similar results were reported for scale collagens from swim bladders of yellowfin tuna [10]; skin of channel catfish [38]; croceine and redlip croakers [23]; and cartilages of scalloped hammerhead, red stingray, and skate [15]. It was reported that the protein molecules had a net positive charge, and protein solubility is increased by repulsion forces between the charged side chains when the pH is not equal to the *pI*. In contrast, when the pH was equal or close to *pI*, the total net charge of the protein molecules approached zero and resulted in precipitation [23,38]. Therefore, the *pI*s of ASC-SB and PSC-SB were approximately 7 and 8 respectively, which was in accordance with the previous report that the *pI* of collagen varies from 6 to 9 [15,39]. At the same pH tested, PSC-SB had higher solubility than ASC-SB, which could be due to the predominance of weaker bonds, and/or lower cross-linking degree of PSC. These solubility behaviors of ASC-SB and PSC-SB with pH and NaCl concentration changes might play an important role in their extraction process.

### 2.7. Zeta Potential

**Figure 6:**
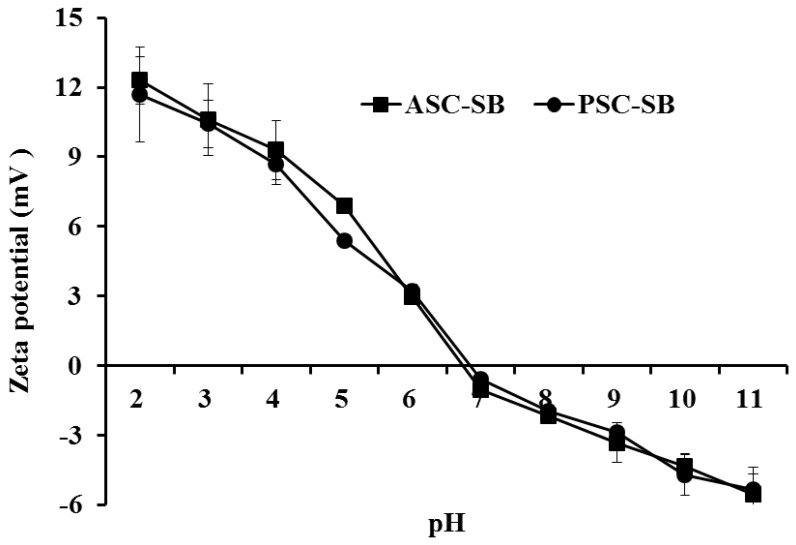
Zeta potential of ASC-SB and PSC-SB from swim bladders of miiuy croaker (*M. miiuy*) at different pH levels. All data are presented as the mean ± SD of triplicate results.

Zeta potential is a key indicator of the stability of colloidal dispersions, which is the potential difference between the dispersion medium and the stationary layer of fluid attached to the dispersed particle [40]. Macromolecules with a high zeta potential have low propensity to form aggregagtes. In contrast, attractive forces among particles may be stronger than repulsion forces when the zeta potential of the particle is close to zero, which would lead to the formation of aggregates [41]. The zeta potential values of the ASC-SB and PSC-SB at pH values ranging from 2 to 11 are shown in Figure 6. The data indicated that ASC-SB and PSC-SB were positively charged at pH values ranging from 2 to 6 and negatively charged at pH values ranging from 7 to 11. The zeta potential data revealed their potential values and *pI*s of ASC-SB and PSC-SB to be 6.74 and 6.85 respectively when their zeta net charges were zero, which is consistent with the result obtained in effect of pH solubility that the *pI*s of ASC-MC and PSC-MC were about pH 7. The difference in zeta potential values between ASC-SB and PSC-SB might be due to the removal of PSC telopeptides by pepsin. Collagen from various fish skins had different zeta potential values, such as ASC and PSC from the swim bladders of yellowfin tuna (6.05 and 5.93 respectively), ASC from scales and skin of tilapia (6.82 and 6.42 respectively) [5], ASC and PSC from skin of loach (6.42 and 6.51 respectively) [42], ASC from skin and swim bladder of seabass (6.46 and 6.64 respectively) [21], ASC and PSC from skin of brown banded bamboo shark (6.21 and 6.56 respectively) [43], and ASC and PHSC from skin of channel catfish (5.34 and 5.42 respectively) [38]. The differences in collagen zeta potential values and *pI*s might be due to amino acid sequences and distribution of amino acid residues.

### 2.8. Collagen Ultrastructure

**Figure 7:**
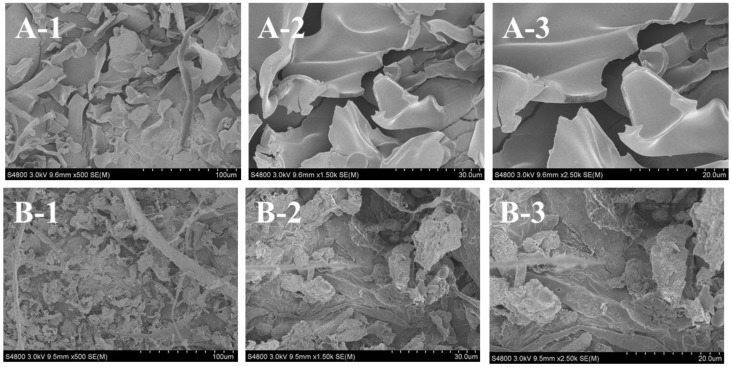
SEM images of ASC-SB and PSC-SB from swim bladders of miiuy croaker (*M. miiuy*). (**A**): ASC-SB; (**B**): PSC-SB. 1: (×500); 2: (×1500); 3: (×2500).

Ultrastructure and surface area of collagen are important to evaluate its potential applications in biomedicine and biomedicine engineering [20,27]. SEM ultrastructure of ASC-SB (Figure 7A) and PSC-SB (Figure 7B) from swim bladders of miiuy croaker were observed at ×500, ×1500, and ×2500. ASC-SB and PSC-SB were like soft white sponge with loose, uniform, and regular alveolate structure as observed by the naked eye, and the pore size increased at higher water content during preparation. Therefore, freeze-drying technology is an advantageous process for developing homogenous porous collagen. In addition, ASC-SB and PSC-SB presented irregular, dense, sheet-like films linked by random-coiled filaments under SEM (Figure 7), and the surface was partially wrinkled, possibly because of dehydration during lyophilizing. The fibrillar structures of ASC-SB and PSC-SB with inter-connected network pore configurations were similar to those of collagens from skin and bone of spanish mackerel [11], gutted silver carp [27], swim bladder of carp [20], and skin of Amur sturgeon [44]. In addition, there were more sheet-like film structures present for ASC-SB than PSC-SB at the same concentration (5% w/v), which suggest that there are some differences in the primary structures between the two. Previous reports suggested that collagen with fibrillary and sheet like film structures with interconnectivity could be used in new tissue formation, cell seeding, growth, wound healing, gene expression, and mass transport and migration [27]. In general, when collagen is employed as a drug carrier, the uniform and regular structure is propitious for a well-proportioned drug distribution. Therefore, the microscopic structures of ASC-SB and PSC-SB indicate that they may be suitable biomaterials for a drug carrier system.

### 2.9. Antioxidant Activity

At present, collagens derived from marine organisms have attracted broad attention in pharmaceutical and cosmeceutical industries because of their antioxidant, antimicrobial, and anti-aging activities [45]. The radical scavenging activity is an important property for the cosmeceutical products aimed at the prevention of photaging and ultraviolet damage [46]. Therefore, four kinds of radical (2,2-diphenyl-1-picrylhydrazyl (DPPH) radical, hydroxyl radical, superoxide anion radical, and 2, 2′-azino-bis-3-ethylbenzothiazoline-6-sulfonic acid (ABTS) radical) scavenging assays were employed to evaluate the antioxidant activities of ASC-SB and PSC-SB from swim bladders of miiuy croaker.

**Figure 8:**
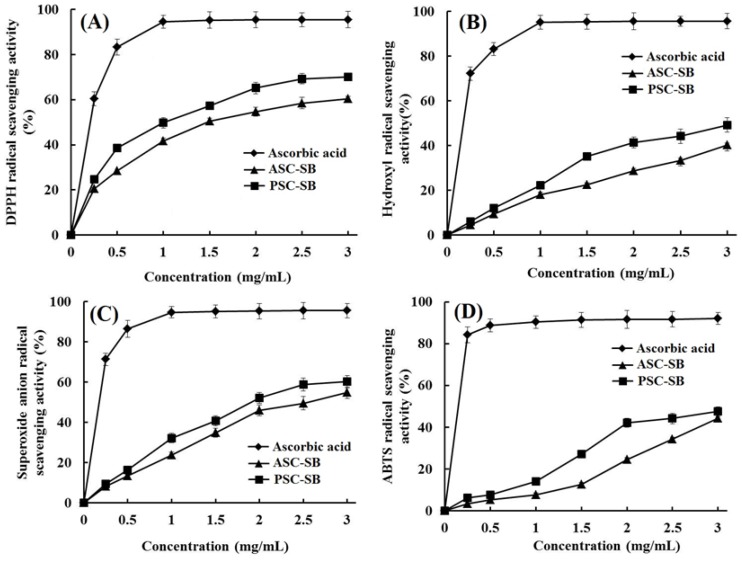
DPPH radical (**A**), hydroxyl radical (**B**), superoxide anion radical (**C**), and ABTS radical (**D**) scavenging activities of ASC-SB and PSC-SB from swim bladders of miiuy croaker. All data are presented as the mean ± SD of triplicate results.

As shown in Figure 8, ASC-SB and PSC-SB from swim bladders of miiuy croaker could scavenge DPPH radical, hydroxyl radical, superoxide anion radical, and ABTS radical in a dose-dependent manner. At all concentrations, the radical scavenging activity of PSC-SB was higher than that of ASC-SB. In addition, ASC-SB and PSC-SB showed higher scavenging capability on DPPH radical (Figure 8A), hydroxyl radical (Figure 8), and superoxide anion radical (Figure 8C) than that of ABTS radical (Figure 8D). As an important evaluation index, the radical scavenging activities of collagens from different marine resources have been reported. Pal and Suresh reported that the DPPH radical scavenging activity range was 9–24% for ASC and 6–20% for PSC at concentrations ranging from 0.2 to 1.0 mg/mL; in addition, both ASC and PSC of catla and rohu showed comparatively higher scavenging activity towards peroxyl radical in a dose-dependent manner at concentrations ranging from 80 to 400 μg/mL [47]. Jeevithan et al. found that ASC and PSC from the cartilage of silvertip shark have strong DPPH radical scavenging activity and reducing power ability [13,48]. It is speculated that the antioxidant mechanism of collagens involves the ability to reduce hydroperoxides, inactivate reactive oxygen species, chelate pro-oxidative transition metals, enzymatically eliminate specific oxidants, and scavenge free radicals [45,46].

In the human body, an imbalance between free radical and antioxidants leads to skin damage, inflammation, cancer, and neuron-related diseases. HO• could almost destroy all biological macromolecules, such as proteins and enzymes, carbohydrates, lipids (lipid peroxidation), and nucleic acids (mutations) [8]. Superoxide anion radical is the most common free radical generated in vivo. It can produce hydrogen peroxide and hydroxyl radical through dismutation and other types of reactions which are the source of free radicals formed in vivo. Both superoxide anion radical and its derivatives are cell-damaging, which can cause damage to DNA and cellular membranes [11]. Therefore, the damage caused by the highly reactive free radicals is widely accepted as the primary reason for skin aging [45]. Thus, ASC-SB and PSC-SB might have a high antioxidant activity similar to that of superoxide dismutase (SOD) and could be used as hydroxyl radical and superoxide radical scavenger in cosmeceutical products for reducing or eliminating radical damage in skin aging.

Skin acts as a chemical and physical barrier to protect the body against foreign pollutants including harmful chemicals, ultraviolet (UV) light exposure, and temperature changes. Photoaging is characterized by changes in the skin due to exposure of ultraviolet (UV)-A (400 to 320-nm wavelength) and UV-B (320 to 290-nm wavelength) light. At present, considerable attention has been paid to the use of fish-derived collagen, gelatin, and peptides for protecting skin from photoaging due to their biocompatibility, penetration ability, excellent antioxidant activity, and skin-repairing ability [34]. Zhuang et al. reported that collagen and collagen hydrolysate from jellyfish could both significantly protect the skin lipids and collagens from UV radiation, and stimulate the synthesis of skin collagen [49]. Collagen polypeptide fractions (CP1 (2 kDa < Mr < 6 kDa) and CP2 (Mr < 2 kDa)) from cod skin could protect skin structures against UV-induced wrinkle formation and destruction, in addition to providing good moisture absorption and retention properties [50]. Chen et al. reported that oral administration of gelatin hydrolysate from the Pacific cod skin could suppress UV radiation-induced damage to the skin by inhibiting the depletion of endogenous antioxidant enzyme activity, and by suppressing the expression of nuclear factor-κB (NF-κB) and NF-κB-mediated expression of pro-inflammatory cytokines [51]. Overall, marine fish-derived collagens, gelatin, and peptides could protect skin from photoaging through alleviating UV-induced abnormal changes of antioxidant defense systems, repairing endogenous collagen and elastin protein fibers, and decreasing the loss of moisture and lipids.

Therefore, the present finding suggested that ASC-SB and PSC-SB from swim bladders of miiuy croaker have high radical scavenging activity and may be applied in cosmeceutical products for preventing photoaging and ultraviolet damage in biological systems.

## 3. Experimental Section

### 3.1. Chemicals and Reagents

The swim bladders of miiuy croaker (*M. miiuy*) were obtained from Zhejiang Hailisheng Group Co. Ltd., in Zhoushan City, Zhejiang Province of China. High molecular weight markers, and type I collagen from calf skin (CSC) were used as the standards and obtained from Sigma-Aldrich (St. Louis, MO, USA). All other reagents used were of analytical grade.

### 3.2. Extraction of Collagen from Swim Bladders of Miiuy Croaker

Extraction of collagen from swim bladders of miiuy croaker was performed using the method of Kaewdang et al. with a slight modification [10]. Non-collagenous proteins of the swim bladders were removed using 0.15 M NaOH at a swim bladders/alkali solution ratio of 1:15 (w/v), and the mixture was stirred for 24 h at 4 °C with a change of the alkali solution every 4 h. After that, the swim bladders were washed with cold distilled water and defatted by 15% (v/v) butyl alcohol with a sample/solution ratio of 1:20 for 48 h with a change of solution every 12 h. Defatted sample was washed with 10-fold volume of cold distilled water three times.

The pretreated sample was soaked in 0.5 M acetic acid with a swim bladder to solvent ratio of 1:15 (w/v) for 48 h at 4 °C with continuous stirring. The mixture was filtered with two layers of cheesecloth. The collagen in the supernatant was precipitated by adding NaCl to a final concentration of 2.5 M. The resultant precipitate was collected by centrifugation at 20,000× *g* for 30 min at 4 °C. The pellet was dissolved in a minimum volume of 0.5 M acetic acid and dialyzed against 10-fold volume of 0.1 M acetic acid for 12 h. After that, it was dialyzed against 15-fold volume of distilled water for 48 h, and distilled water was changed every 12 h. The resulting dialysate was lyophilized and referred to as acid soluble collagen (ASC-SB).

The residue from ASC-SB extraction was further suspended in 10-fold volume of 0.5 M acetic acid and porcine pepsin (20 U/g residues) was added. The mixtures were continuously stirred at 4 °C for two days. After that, the supernatant was obtained in the same manner for ASC-SB preparation including precipitation, dialysis, and lyophilisation. The obtained collagen was referred to as pepsin soluble collagen (PSC-SB).

The yields of ASC-SB and PSC-SB were calculated on the dry swim bladder weight.

Yield (%) = (Weight of freeze dried collagen (g)/Weight of initial dry swim bladder (g)) × 100

### 3.3. Proximate Analysis

Moisture, ash, and fat contents of swim bladder and collagens were determined using the methods of the Association of Official Agricultural Chemists (AOAC) method with the method numbers of 950.46B, 920.153, and 960.39 (a), respectively [52]. Protein content was measured using the Kjeldahl method and an auto protein analyzer (Kjeltec 2400 auto-analyzer, Hillerød, Denmark). The converting factor of 6.25 was used for calculation of protein content [15].

### 3.4. Amino Acid Analysis

Amino acid analysis was measured according to the methods described by Chi et al. [15]. Tested samples were hydrolyzed in 6 M HCl at 110 °C for 24 h, and the hydrolysates were concentrated and the residues were dissolved in 25 mL citric acid buffer solution. An aliquot of 0.05 mL was applied to an automated amino acid analyzer of HITACHI 835-50 (Hitachi High-Technologies Corporation, Tokyo, Japan). Then the degrees of Pro hydroxylation (%) and Lys hydroxylation (%) were calculated as

Degrees of Pro hydroxylation (%) = Hydroxyproline Content/(Hydroxyproline Content + Proline Content) × 100%

Degrees of Lys hydroxylation (%) = Hydroxylysine Content/(Hydroxylysine Content + Lysine Content) × 100%

### 3.5. Electrophoretic Pattern

Electrophoretic patterns of collagens were determined using the previous method [11], employing a 7.5% resolving gel and 4% stacking gel. Collagen samples were suspended in 5% (w/v) SDS prior to incubation at 85 °C for 1 h. The mixture was centrifuged at 5000× *g* for 10 min at room temperature to remove undissolved debris. The samples (about 20 μL) were mixed with the sample loading buffer (60 mM Tris-HCl, pH 8.0, containing 25% glycerol, 2% SDS, 0.1% bromophenol blue) at a ratio of 4:1 (v/v) in the presence of β-ME, then applied to sample wells, and electrophoresed in an electrophoresis instrument (AE-6200, ATTO Corporation, Tokyo, Japan). The electrophoresis was carried out for about 4 h at a constant voltage of 100 V. After electrophoresis, gel was stained with 0.1% (w/v) Coomassie blue R-250 in 45% (v/v) methanol and 10% (v/v) acetic acid. High molecular weight protein markers (Shanghai Institute of Biochemistry, the Chinese Academy of Sciences, Shanghai, China) were used to estimate the molecular weight of proteins.

### 3.6. Spectral Properties of Collagen

#### 3.6.1. UV Absorption Spectrum

The UV adsorption spectra of ASC-SB and PSC-SB were recorded using the method of Yu et al. [16], using a spectrophotometer (UV-1800, Mapada Instruments Co., Ltd., Shanghai, China) from 200 to 400 nm. The sample was prepared by dissolving the collagen in 0.5 moL/L acetic acid solution with a sample to solution ratio of 1:1 000 (w/v).

#### 3.6.2. FTIR Spectral Analysis

The IR spectra of ASC-SB and PSC-SB were recorded in KBr disks with a FTIR spectrophotometer (Nicolet 6700, Thermo Fisher Scientific Inc., Waltham, MA, USA). The mixture at a sample to potassium bromide (KBr) ratio of 1:100 (w/w) was pressed into a disk for spectrum recording. The IR spectra in the range of 4000–400 cm^−1^ with automatic signal gain were collected in 32 scans at a resolution of 4 cm^−1^ and were ratioed against a background spectrum recorded from the clean empty cell.

### 3.7. Viscosity

Collagen viscosity was measured using the previous method [9]. All the samples were dissolved in deionized water with the vibration of THZ-100 shaker (Shanghai Yiheng Technical Co., Ltd., Shanghai, China), to obtain a concentration of 0.6% (w/v), and 500 mL solutions were subjected to viscosity measurement using a NDJ-8S viscometer (Jingtian Instruments Co., Ltd., Shanghai, China). The sample solutions were heated from 4 to 40 °C with a heating rate of 4 °C/min, and the solution was held for 30 min prior to viscosity determination at the designated temperature. The relative viscosity was calculated in comparison with that obtained at 4 °C. Denaturation temperature (T_d_) was defined as the temperature at which fractional viscosity was 0.5.

### 3.8. Solubility

Effect of pH and NaCl concentration on the collagen solubility was measured using the previous method [9]. Collagen solutions (3.5 M) were prepared using 0.5 M acetic acid solution and stirred for 24 h at 4 °C. The solutions were centrifuged at 10,000× *g* for 15 min at 4 °C, and the resulting supernatants were used for measuring solubility of collagen.

#### 3.8.1. Effect of pH on Solubility

Sample solution (8 mL) was transferred to a 50 mL centrifuge tube and the pH was adjusted with either 6 M NaOH or 6 M HCl to obtain the final pH ranging from 1 to 11. The volume of solution was made up to 10 mL by deionized water previously adjusted to the same pH as the sample solution. The solution was centrifuged at 15,000× *g* for 60 min at 4 °C. For all the samples, protein content in the supernatant was measured. Then the relative solubility was calculated in comparison with that obtained at the pH giving the highest solubility.

#### 3.8.2. Effect of NaCl on Solubility

Sample solution (5 mL) was mixed with 5 mL of NaCl in 0.5 M acetic acid at various concentrations to give the final concentrations of 0, 1, 2, 3, 4, 5, and 6%. The mixture was stirred continuously at 4 °C for 30 min, followed by centrifuging at 15,000× *g* for 60 min at 4 °C. Protein content in the supernatant was measured and the relative solubility was calculated as previously described.

### 3.9. Zeta Potential

ASC-SB and PSC-SB were dissolved in 0.05 M acetic acid to a final concentration of 0.2 mg/mL and incubated at 4 °C for 48 h. The zeta potential of ASC-SB and PSC-SB was determined using a NanoBrook Omni zeta potential analyzer (Brookhaven, Inc., Brookhaven, MS, USA) as reported by Chen et al. [5]. The pH of the samples (20 mL) was adjusted to a pH range (2–11) with 1 M KOH and 1 M HCl. The *pI*s of ASC-SB and PSC-SB showed that the pH value that resulted in a zero zeta potential.

### 3.10. Collagen Ultrastructure

The morphological characteristics of ASC-SB and PSC-SB were studied by scanning electron microscopy (SEM) using Hitachi TM-1000 (Tokyo, Japan). Collagen was redissolved in 0.5 M acetic acid at a concentration of 5% (w/v), followed by dialyzing against distilled water. The collagen was lyophilized in a freeze dryer (EYELA FD-1000, Tokyo Rikakikai Co., Ltd., Tokyo, Japan) and the sample was sputter coated for 90 s with gold using a JFC-1200 (JEOL Ltd, Tokyo, Japan) fine coater. The morphologies of the electro spun fibers and membrane were observed using Hitachi TM-1000 (Hitachi High-Technologies Corporation, Tokyo, Japan).

### 3.11. Antioxidant Activity

The DPPH radical, hydroxyl radical, superoxide anion radical, and ABTS radical scavenging activity of ASC-SB and PSC-SB was performed according to previously reported methods [8,53].

#### 3.11.1. DPPH Radical Scavenging Activity

Two milliliters of deionized water containing different concentrations of samples were placed in cuvettes, and then 500 μL of ethanol solution of DPPH (0.02%) and 1.0 mL of ethanol were added. Both a control, containing DPPH solution without sample, and a blank in which DPPH solution was substituted with ethanol, were prepared. In blank, DPPH solution was substituted with ethanol. The antioxidant activity of the sample was evaluated by the inhibition percentage of DPPH radical with the equation

DPPH radical scavenging activity (%) = (A_0_ + A′ − A)/A_0_ × 100%

where A was sample absorbance rate; A_0_ was the absorbance of control group; A′ was the blank absorbance.

#### 3.11.2. Hydroxyl Radical Scavenging Activity

In this system, hydroxyl radicals are generated by the Fenton reaction. Hydroxyl radicals can oxidize Fe^2+^ into Fe^3+^, and only Fe^2+^ can combine with 1,10-phenanthroline to form a red compound (1,10-phenanthroline-Fe^2+^) with the maximum absorbance at 536 nm. The concentration of hydroxyl radical is reflected by the degree of decolorization of the reaction solution. Briefly, 1,10-phenanthroline solution (1.0 mL, 1.865 mM) and the sample (2.0 mL) were added into a screw-capped tube and mixed. The FeSO_4_·7H_2_O solution (1.0 mL, 1.865 mM) was then pipetted into the mixture. The reaction was initiated by adding 1.0 mL H_2_O_2_ (0.03% v/v). After being incubated at 37 °C for 60 min in a water bath, the absorbance of the reaction mixture was measured at 536 nm against a reagent blank. The reaction mixture without any antioxidant was used as the negative control, and mixture without H_2_O_2_ was used as the blank. The hydroxyl radical scavenging activity was calculated by the formula:

Hydroxyl radical scavenging activity (%) = [(A_s_ − A_n_)/(A_b_ − A_n_)] × 100%

where A_s_, A_n_, and A_b_ were the absorbance values determined at 536 nm of the sample, the negative control, and the blank after reaction, respectively.

#### 3.11.3. Superoxide Anion Radical Scavenging Activity

In the experiment, superoxide anions were generated in solutions containing 1 mL of nitrotetrazolium blue chloride (NBT) (2.52 mM), 1 mL of NADH (624 mM), and 1 mL of different concentrations of samples. The reaction was initiated by adding 1 mL of phenazine methosulfate (PMS) solution (120 μg) to the reaction mixture. The absorbance was measured at 560 nm against the corresponding blank after 5 min incubation at 25 °C. The capacity of scavenging the superoxide anion radical was calculated using the equation

Superoxide anion radical scavenging activity (%) = [(A_control_ − A_sample_)/A_control_] × 100%

where A_control_ was the absorbance without sample and A_sample_ was the absorbance with sample.

#### 3.11.4. ABTS Radical Scavenging Activity

The ABTS radical cation was generated by mixing ABTS stock solution (7 mM) with potassium persulphate (2.45 mM). Mixture was left in the dark at room temperature for 16 h. The ABTS radical solution was diluted in 5 mM phosphate buffered saline (PBS) pH 7.4, to an absorbance of 0.70 ± 0.02 at 734 nm. One milliliter of diluted ABTS radical solution was mixed with one milliliter of different concentrations of samples. Ten minutes later, the absorbance was measured at 734 nm against the corresponding blank. The ABTS radical scavenging activity of samples was calculated using the equation

ABTS radical scavenging activity (%) = [(A_control_ − A_sample_)/A_control_] × 100%,

where A_control_ was the absorbance without sample and A_sample_ was the absorbance with sample.

### 3.12. Statistical Analysis

All experiments were carried out in triplicate. An ANOVA test using the software of SPSS 19.0 (Statistical Program for Social Sciences, SPSS Corporation, Chicago, IL, USA) was applied to compare the average values of each treatment. Duncan’s multiple range test (*p* < 0.05) was used to measure the significant differences between the parameters means.

## 4. Conclusions

In this report, acid-soluble collagen (ASC-SB) and pepsin-soluble collagen (PSC-SB) from swim bladders of miiuy croaker (*M. miiuy*) were isolated with yields of 1.33 ± 0.11% and 8.37 ± 0.24%. Physicochemical property of ASC-SB and PSC-SB confirmed that they were mainly composed of type I collagen with the triple helical structures. In addition, ASC-SB and PSC-SB had a much lower T_d_ as compared to mammalian collagen due to low imino acid contents (196.7 and 199.5.9 residues/1000 residues), which suggested that ASC-SB and PSC-SB were unstable compared to collagens from mammalian sources. In addition, ASC-SB and PSC-SB could scavenge DPPH radical, hydroxyl radical, superoxide anion radical, and ABTS radical in a dose-dependent manner. The present results provide helpful information for preparation of collagens from swim bladders of miiuy croaker and their further application in food and pharmaceutical industries.

## Figures and Tables

**Table 1:** Chemical compositions of swim bladders, ASC-SB, and PSC-SB from miiuy croaker (*M. miiuy*)

Sample	Proximate Compositions (g/100 g Dry Weight)				Yield on Dry Weight Basis (%)
	Moisture	Protein	Fat	Ash	
Swim bladders	78.83 ± 2.37 ^a^	19.17 ± 0.15 ^a^	1.63 ± 0.11 ^a^	0.35 ± 0.06 ^a^	
ASC-SB	4.71 ± 0.28 ^b^	94.25 ± 2.57 ^b^	0.43 ± 0.12 ^b^	0.96 ± 0.15 ^b^	1.33 ± 0.11
PSC-SB	4.31 ± 0.29 ^b^	95.02 ± 3.04 ^b^	0.31 ± 0.08 ^b^	0.62 ± 0.12 ^c^	8.37 ± 0.24

All values are mean ± standard deviation (SD) (n = 3). ^a–c^ Values with same letters indicated no significant difference (*p* > 0.05).

**Table 2:** Amino acid composition of CSC, ASC-SB, and PSC-SB from swim bladders of miiuy croaker (*M. miiuy*) (residues/1000 residues)

Amino Acid	ASC-SB	PSC-SB	CSC
Hydroxyproline	89.5	87.6	95.1
Glutamine/glutamic acid	44.6	39.4	45.7
Threonine	21.9	21.7	18.4
Serine	28.7	27.9	33.2
Glutamine/glutamic acid	90.3	84.9	75.9
Proline	107.2	111.9	121.5
Glycine	320.5	333.6	330.6
Alanine	96.6	95.1	119.7
Cysteine	0.2	0.4	0.0
Valine	23.6	32.9	21.5
Methionine	6.6	5.2	6.1
Isoleucine	11.8	13.3	11.4
Leucine	34.8	26.9	23.4
Tyrosine	7.7	2.3	3.7
Phenylalanine	26	23.4	3.3
Hydroxylysine	6.7	6.2	7.7
Tryptophan	0.0	0.0	0.0
Lysine	32.7	24.2	26.5
Histidine	9.3	8.1	5.3
Arginine	41.3	55	51.0
Total	1000.0	1000.0	1000.0
Imino acid (Proline + Hydroxyproline)	196.7	199.5	216.6
